# Prevention of Alcohol-Related Crime and Trauma (PACT): brief interventions in routine care pathway – a study protocol

**DOI:** 10.1186/1471-2458-13-49

**Published:** 2013-01-18

**Authors:** Rama Jayaraj, Megan Whitty, Mahiban Thomas, David Kavangh, Didier Palmer, Valerie Thomson, Carolyn Griffin, Luke Mayo, Peter D’Abbs, Tricia Nagel

**Affiliations:** 1 Wellbeing and Preventable Chronic Diseases Division, Menzies School of Health Research and School of Psychological and Clinical Sciences and Charles Darwin University, Darwin, Northern Territory, Australia; 2 Wellbeing and Preventable Chronic Diseases Division, Menzies School of Health Research, Darwin, Northern Territory, Australia; 3 Department of Head and Neck Surgery, Royal Darwin Hospital, Darwin, Northern Territory, Australia; 4 Institute of Health & Biomedical Innovation and School of Psychology & Counselling, Queensland University of Technology, Kelvin Grove, QLD, Australia; 5 Emergency Department, Royal Darwin Hospital, Darwin, Northern Territory, Australia

**Keywords:** Alcohol-related trauma, Screening, Brief intervention, Dissemination

## Abstract

**Background:**

Globally, alcohol-related injuries cause millions of deaths and huge economic loss each year . The incidence of facial (jawbone) fractures in the Northern Territory of Australia is second only to Greenland, due to a strong involvement of alcohol in its aetiology, and high levels of alcohol consumption. The highest incidences of alcohol-related trauma in the Territory are observed amongst patients in the Maxillofacial Surgery Unit of the Royal Darwin Hospital. Accordingly, this project aims to introduce screening and brief interventions into this unit, with the aims of changing health service provider practice, improving access to care, and improving patient outcomes.

**Methods:**

**Establishment of Project Governance:** The project governance team includes a project manager, project leader, an Indigenous Reference Group (IRG) and an Expert Reference Group (ERG).

**Development of a best practice pathway:** PACT project researchers collaborate with clinical staff to develop a best practice pathway suited to the setting of the surgical unit. The pathway provides clear guidelines for screening, assessment, intervention and referral.

**Implementation:** The developed pathway is introduced to the unit through staff training workshops and associate resources and adapted in response to staff feedback.

**Evaluation:** File audits, post workshop questionnaires and semi-structured interviews are administered.

**Discussion:**

This project allows direct transfer of research findings into clinical practice and can inform future hospital-based injury prevention strategies.

## Background

Half of alcohol-attributable deaths are a result of injury worldwide [1]. Alcohol-related harm is a major cause of mortality and morbidity in Australia, causing around 3,000 deaths and 65,000 hospitalisations every year [2]. Alcohol-related injuries are more commonly caused by heavy drinking than by people with severe alcohol dependence, and strong links exist between alcohol-related trauma, crime and binge drinking [3].

The most recent national survey of drug use estimates that one in five Australians drink at a level that puts them at risk of short-term harm at least once a month [4], and rates in its Northern Territory (NT) are especially high. Indigenous Australians who constitute a substantial proportion of residents of NT (32%) and they are six times more likely to drink at high-risk levels than non-Indigenous Australians [5]. In consequence, Indigenous Australians are also at greater risk of alcohol-related harms than other Australians. Harms associated with high-risk alcohol consumption in Indigenous Australians include family conflict, domestic violence and assaults [6,7], and alcohol is the leading cause of injury among Indigenous Australians, followed by intimate partner violence [8]. In fact, rates of death from exclusively alcohol-related conditions are almost eight times greater for Australian Indigenous males than for non-Indigenous males and 16 times greater for Indigenous females than for non-Indigenous females among residents of Western Australia, South Australia and Northern Territory [9]. The percentage of alcohol-related deaths among young Indigenous Australians aged 15–24 is almost three times higher than for their non-Indigenous counterparts [9].

Alcohol-related violence is the most common cause of hospital admission for injury in the NT [10], accounting for 38% of the total injury admissions for Indigenous people. Further, it has been reported that most of the assaults against women in remote NT communities are perpetrated by a drunken husband or other family member. Alcohol-related facial trauma is common, with an estimated 350 cases per year admitted to the Maxillofacial Surgery Unit of the Royal Darwin Hospital (RDH) [11,12]. Many of these admissions, approximately 80% are of Indigenous people [12]. Therefore, there is an urgent need for an effective and culturally appropriate intervention to address binge drinking and alcohol-related harm in this group. In the general population, screening and brief counselling can reduce high-risk alcohol consumption and alcohol-related assaults associated with binge drinking, but more research is needed on alcohol-related trauma among the NT Indigenous people. ‘Motivational care planning’ (MCP) is a broad-based motivational intervention to improve health and wellbeing of Indigenous Australians. In early research, it demonstrated acceptability and an ability to engage participants [13], and it resulted in significant improvements in wellbeing, substance use and self-management [13,14]. This project adapts and applies MCP to inpatients with alcohol-related facial injuries.

## Methods

### Aim

The PACT project aims to introduce routine screening and brief intervention by staff of the Maxillofacial Surgery Unit at Royal Darwin Hospital (RDH), in order to raise awareness of at-risk drinking and prevent recurrent injury in inpatients with alcohol-related facial injuries.

#### This project aims to answer the question

Will introduction of screening and brief interventions change health service provider practice and reduce alcohol-related injuries secondary to assault?

We predict that a participatory action approach to implementing a best practice pathway to referral and treatment for high-risk alcohol users admitted to the Maxillofacial Surgical Unit with injuries will change health service provider practice and reduce alcohol-related injury.

### Research plan

This 18-month project introduces screening and brief interventions for high-risk drinkers admitted to hospital with facial trauma and evaluates the implementation of a best practice pathway. The project transfers skills and resources to hospital staff to support delivery of best practice and to evaluate progress through continuous quality improvement strategies.

### Establishment of Project Governance

An Indigenous Reference Group and an Expert Reference Group oversee the project. The IRG comprises senior urban- and community-based Australian Indigenous people and is established through Menzies School of Health Research. The ERG includes senior representatives from the Maxillofacial Surgery Unit and the NT Department of Health. The research team consists of a project leader and project manager from Menzies School of Health Research, senior representatives from the Maxillofacial Surgery Unit and Indigenous researchers at Menzies. The day-to-day management of the project is the responsibility of the project leader and project manager, in collaboration with the Indigenous research officers. The research team and ERG formally meet by teleconference or face-to-face every three to six months.

### Development of best practice pathway

**Figure 1:**
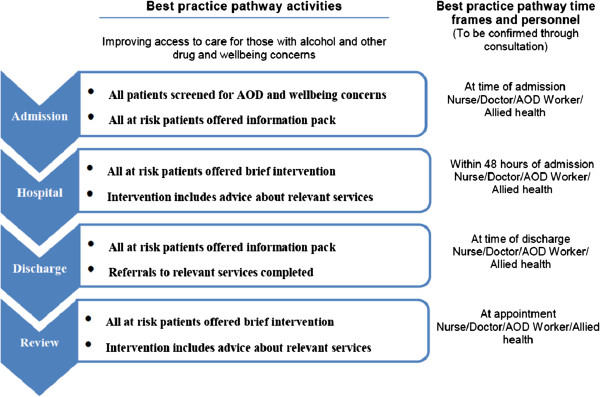
PACT pathway of study.

A tailored best practice pathway suited to the setting of the surgical unit is developed in collaboration with hospital staff, following exploration of current systems (Figure 1). The pathway includes four key activities: (1) Brief screening of all admissions, (2) An information booklet for those at risk, (3) Referral to appropriate services for those at risk, (4) Delivery of a culturally-adapted brief intervention. Staff are trained to apply the best practice pathway to all patients.

All relevant services in Darwin are contacted via email and phone, informed of the project aims and invited to participate. Engagement of community services includes visiting the organisation, understanding specific referral processes and exploring the capacity of external services to provide in-house services for potential clients in the hospital. Representatives from the various agencies present at PACT workshops to inform and educate hospital staff about available services for patients with substance abuse problems and/or wellbeing concerns. A pamphlet is developed containing contact information and a brief synopsis of Alcohol and Other Drugs (AOD), mental health and domestic violence services both within and outside the hospital system

### Implementation of best practice pathway

Implementation is multi-faceted, and includes information and consultation meetings with staff and community service providers, staff training workshops, key informant interviews, feedback sessions and the introduction of relevant resources (available online and in hard copy form). The implementation of the best practice pathway includes three key activities: (1) training to familiarise staff with the tools, (2) workshops to train staff in delivery of brief interventions, (3) a feedback workshop to review the effectiveness of implementation through training evaluations, file audits and interviews. The chosen brief intervention is based on findings of a project conducted by the Australian Integrated Mental health initiative (AIMhi) in the NT (Nagel et al., 2009).

#### Information and consultation meetings with staff and community service providers

Consultation meetings are conducted with hospital staff and community service providers to inform the development of best practice protocols for detection and treatment of patients who demonstrate high-risk drinking. These meetings aim to improve understanding of the current strategies and practical issues surrounding implementation in the hospital setting.

#### Staff training workshops

Up to six PACT training workshops, involving up to 50 hospital-based service providers, are conducted. The workshops provide information about screening, referral services and brief interventions. A post-workshop participant evaluation questionnaire is collected and results analysed. The questionnaire incorporates ordinal scales and open-ended questions. Participants are asked about knowledge and confidence in screening, brief intervention and referral for at-risk drinkers. Knowledge and confidence are rated on a scale from 1 (not much /not confident) to 9 (a lot /very confident). Participants are also asked to rate how interesting and useful the workshop was on a scale from 1 (not at all) to 4 (very). The questionnaire includes a section for attendees to comment on their experience and state whether they would change their practice as a result of the workshop. A joint feedback workshop in the last three months of the study will report on the key findings from staff training activities and file audits over the course of the project.

#### Resource development

Brief intervention resources and a best practice protocol manual are prepared. Tools for ongoing continuous quality improvement are made available to the surgery unit.

#### Feedback

Feedback of interview responses by key informants and the results of the file audits allow opportunity for refinement of the care pathway, goal setting, further training and dissemination. Project findings will be presented at relevant conferences within Australia and results are to be published in appropriate scientific journals, particularly those that target hospital care of high-risk drinkers.

### Evaluation of the best practice pathway process

#### Process evaluation

The process activities include evaluating: (1) number of workshops held and workshop content and format, staff trained, (2) number and type of staff attending workshops, and (3) number and type of training and education resources developed.

### Outcome evaluation

Outcome evaluation activities include 1) file audits, and 2) semi structured interviews.

#### File audits

The project team conducts two file audits: one at baseline and one at 9 months, and records the number of admissions related to high-risk drinking, screenings, patients flagged to be ‘at-risk’, brief interventions delivered, information distributed and referrals completed. The review of files allows the project to monitor outcomes. This information is essential for review and feedback for quality improvement and development of care processes.

#### Key informant interviews

The project team conducts a small number of key informant interviews to explore client, family and service provider perspectives on the process. Five key informant interviews with surgical unit staff assess confidence and knowledge as well as challenges and enablers to screening and best practice. Five key informant interviews with patients and families explore their experience of the best practice pathway.

### Selection criteria

There are two target populations: (1) Service providers who care for clients admitted to the Maxillofacial Surgical Unit, and (2) Patients or Clients admitted to Royal Darwin Hospital with facial trauma during the study. Patient participants will need to be at least 18 and able to give informed consent.

### Statistical analysis and sample size

Analysis of file audits and interviews will include descriptive statistics and qualitative data grouped and analysed by theme.

There are two samples: files to be audited and individuals to be interviewed.

1. The files to be audited will include a sample of trauma patients admitted to the Maxillofacial Surgery Unit during the six months prior to commencement of the study and the 9 months of the study from baseline (estimated 160 files). Files are examined for frequency of screening, recorded evidence of brief interventions given for those at risk and documentation of uptake of the new pathway. They will also be examined for client outcomes in terms of wellbeing, alcohol-related medical problems and high-risk drinking.

2. A small sample of client participants and service providers will be interviewed to assess establishment of the new pathway within routine care. We will purposely sample five clients and five service providers to explore enablers and challenges and the client experience. We have chosen this sample size in order to be able to gain some insight into these client and service provider perspectives whilst keeping within the resources and brief time frames of the study.

### Ethical approvals

The study has been granted full ethics approval by the Human Research Ethics Committee of Department of Health and Menzies School of Health Research (HREC-11-1553). Data are accessible to the investigators and support investigation team only. In the audit forms, we will not record identifiable client information such as client’s names or registration numbers. Instead, a code will be used as identifier. This enables checking of data during the cleaning of audit data where necessary. Codes linked with client’s names will be retained by the research team and a copy stored electronically with the rest of the data at Menzies in a separate file accessed only by password.

### Engagement with stakeholders

This project is a partnership between the RDH Maxillofacial Surgery Unit, the Alcohol and Other Drug (AOD) program NT wide, the remote AOD Workforce Program and Menzies School of Health Research. The AOD Workforce Program operates within a number of Aboriginal-controlled and government health centres in urban and remote settings across the NT. The NT Department of Health AOD program, the remote AOD workforce and the RDH surgery unit are key supporting partners. The AOD program assisted in the development of this project outline and is committed to its success. The surgery unit of RDH proposed the project and strongly supports the project’s aims.

## Discussion

### Benefits

The project will implement and evaluate strategies for screening and intervention for reducing the harms associated with alcohol consumption in Indigenous patients in the NT in line with the Aboriginal and Torres Strait Islander Peoples Complementary Action Plan 2003–2009. The main objectives of the action plan are control of supply, management of demand, reduction of harm, early intervention and treatment.

The value of the study includes direct benefit to participants through improved wellbeing, decreased recurrence of injury and less substance misuse. The study benefits the service providers who care for high-risk drinkers who sustain injury by allowing them to provide timely advice and intervention.

Indirect benefit to the broader population is expected by improving strategies to engage and treat individuals with substance abuse concerns, refinement of educational materials, and development of best practice protocols that may be generalised to a range of other settings.

## Competing interests

The authors declare that they have no competing interests.

## Authors’ contributions

RJ, MT and TN were the main contributors to the conceptualisation of the study. DK,MW, DP, Pd, VT, CG contributed significantly to this research proposal, and have read and approved the final manuscript.

## Pre-publication history

The pre-publication history for this paper can be accessed here:

http://www.biomedcentral.com/1471-2458/13/49/prepub
